# Arrhythmia detection by the graph convolution network and a proposed structure for communication between cardiac leads

**DOI:** 10.1186/s12874-024-02223-4

**Published:** 2024-04-27

**Authors:** Bahare Andayeshgar, Fardin Abdali-Mohammadi, Majid Sepahvand, Afshin Almasi, Nader Salari

**Affiliations:** 1 Department of Biostatistics, School of Health, Kermanshah University of Medical Sciences, Kermanshah, 6715847141 Iran; 2 Department of Computer Engineering and Information Technology, Razi University, Kermanshah, 6714967346 Iran; 3 Clinical Research Development Center, Mohammad Kermanshahi, and Farabi Hospitals, Imam Khomeini, Kermanshah University of Medical Sciences, Kermanshah, Iran; 4 Sleep Disorders Research Center, Kermanshah University of Medical Sciences, Kermanshah, 6715847141 Iran

**Keywords:** New structure, Arrhythmic, Electrocardiogram, Graph convolution neural network, Weighted mutual information

## Abstract

One of the most common causes of death worldwide is heart disease, including arrhythmia. Today, sciences such as artificial intelligence and medical statistics are looking for methods and models for correct and automatic diagnosis of cardiac arrhythmia. In pursuit of increasing the accuracy of automated methods, many studies have been conducted. However, in none of the previous articles, the relationship and structure between the heart leads have not been included in the model. It seems that the structure of ECG data can help develop the accuracy of arrhythmia detection. Therefore, in this study, a new structure of Electrocardiogram (ECG) data was introduced, and the Graph Convolution Network (GCN), which has the possibility of learning the structure, was used to develop the accuracy of cardiac arrhythmia diagnosis. Considering the relationship between the heart leads and clusters based on different ECG poles, a new structure was introduced. In this structure, the Mutual Information(MI) index was used to evaluate the relationship between the leads, and weight was given based on the poles of the leads. Weighted Mutual Information (WMI) matrices (new structure) were formed by R software. Finally, the 15-layer GCN network was adjusted by this structure and the arrhythmia of people was detected and classified by it. To evaluate the performance of the proposed new network, sensitivity, precision, specificity, accuracy, and confusion matrix indices were used. Also, the accuracy of GCN networks was compared by three different structures, including WMI, MI, and Identity. Chapman’s 12-lead ECG Dataset was used in this study. The results showed that the values of sensitivity, precision, specificity, and accuracy of the GCN-WMI network with 15 intermediate layers were equal to 98.74%, 99.08%, 99.97% & 99.82%, respectively. This new proposed network was more accurate than the Graph Convolution Network-Mutual Information (GCN-MI) with an accuracy equal to 99.71% and GCN-Id with an accuracy equal to 92.68%. Therefore, utilizing this network, the types of arrhythmia were recognized and classified. Also, the new network proposed by the Graph Convolution Network-Weighted Mutual Information (GCN-WMI) was more accurate than those conducted in other studies on the same data set (Chapman). Based on the obtained results, the structure proposed in this study increased the accuracy of cardiac arrhythmia diagnosis and classification on the Chapman data set. Achieving such accuracy for arrhythmia diagnosis is a great achievement in clinical sciences.

## Introduction

Diagnosis and prevention of diseases are one of the most important goals of medical sciences. One of the most common causes of death worldwide is heart disease, including arrhythmia [1–3]. Cardiac arrhythmia and its type are usually diagnosed using a 12-lead Electrocardiogram (ECG) [4]. Types of arrhythmias include Sinus Bradycardia (SB) and Atrial Tachycardia (AT) with an excessively slow or fast heartbeat, or Premature Ventricular Contraction (AVC) with an irregular rhythm and missing or distorted intervals. The most common and most dangerous type of arrhythmia is Atrial Fibrillation (AFIB). This type of arrhythmia carries the risk of severe heart dysfunction and stroke [5]. Other types of arrhythmias include Sinus Tachycardia (ST), Sinus Irregularity (SI), Supraventricular Tachycardia (ST), Atrial Nodal Reentrant Tachycardia (AVNRT), Atrioventricular Reentrant Tachycardia (AVRT), sinus-atrial-to-atrial tachycardia, and Atrial Wandering Rhythm (AWR).

First, the prediction of heart diseases is very important to prevent mortality. Second, the diagnosis of arrhythmia and its type with ECG requires knowledge and experience and is influenced by individual experience and expertise [3, 6–8]. Third, using traditional statistical methods to predict heart diseases has limitations. Therefore, it is not without reason that nowadays sciences such as artificial intelligence and medical statistics seek to introduce methods and models for correct and automatic diagnosis of cardiac arrhythmia [9–14].

Many studies have used Deep Learning Neural Network (DNN) and Convolution Neural Network (CNN) to detect cardiac arrhythmia [15–30]. In 2020, an artificial neural network model called DNN was proposed by Yıldırım et al. to identify different classes of ECG rhythm [26]. Shaker et al. also proposed a GAN network in 2020 to detect and classify the type of rhythm [31]. In 2020, Yao et al. presented an ATI-CNN network aimed at detecting arrhythmia type based on multi-channel ECG signal, in which 6877 12-lead ECG records were included and 8 types of arrhythmia were finally classified [20]. In 2020, Zhou & Tan presented a method for combining Convolutional Neural Network (CNN) and Extreme Learning Machine (ELM) with the aim of automatic identification and classification of ECG signals [5]. CNN has been used in many studies to detect and classify cardiac arrhythmias. CNN is one of the methods of artificial neural networks in which an operation called convolution is used in its layers. This network performs feature selection simultaneously with network learning and does not require complex data preprocessing [32, 33].

ECG measures the intensity of the electrical current of the heart from different angles; therefore, the cardiac leads in the 12-lead ECG are related to each other. However, in all the mentioned studies, this very important relationship has been neglected. It seems that considering this relationship in neural network adjustment increases the accuracy of arrhythmia detection. To achieve this goal, the Graph Convolution Network (GCN) was used in this study. Unlike CNN or DNN networks, GCNs have the relative advantage of being able to learn the structure. In the GCN network, instead of performing the convolution operation on the images consisting of pixels, this operation is performed on the graph [34]. To implement this network, the data and the structure between them must be designed in the form of a graph; i.e. the graph is formed from nodes and edges. Edges show the connection between nodes, and its information is introduced to GCN by a matrix called adjacency matrix [35].

**Fig. 1:**
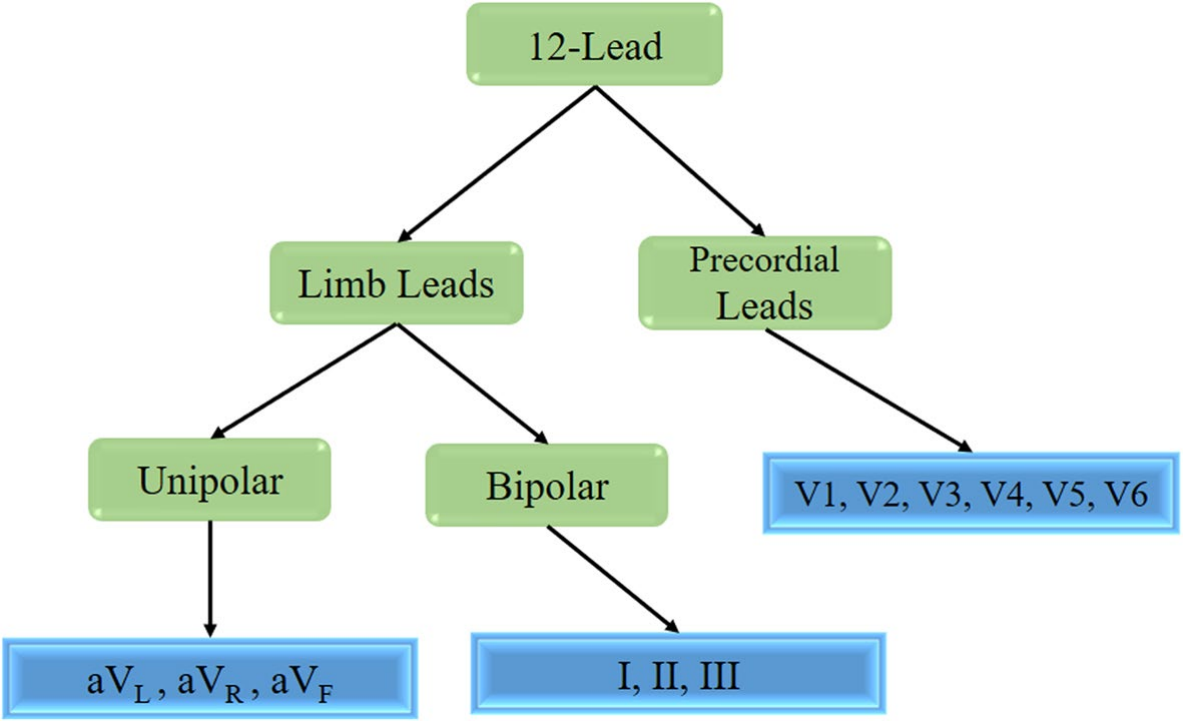
Clustering of heart leads based on the ECG pole

In this study, to convert ECG into a graph, the leads were introduced as nodes, and the relationship between them was introduced as an adjacency matrix by Weighted Mutual Information (WMI). WMI was used as a weighted correlation structure for ECG data. From a statistical point of view, each lead can be considered a time series, and thus a 12-lead ECG can be considered a 12-variable time series [10]. In time series, the measurements have self-correlation, so the correlation between leads cannot be measured by Pearson or Spearman correlation indices, which do not have the independent and identically distributed (iid) assumption. Mutual Information (MI) is a statistical index that measures the linear and non-linear dependencies between two-time series and calculates the common information between them using the definition of entropy [36]. A 2 × 12 matrix is formed for each 12-lead ECG. To form the Weighted Mutual Information (WMI), the MI matrix is weighted. The weight applied to MI is based on the category of cardiac leads (precordial leads, unipolar limb leads, and bipolar limb leads) (Fig. 1).

It contains 6 limp leads and 6 precordial leads. Limb leads include 3 bipolar leads and 3 unipolar leads. Bipolar leads I-II-III have two positive and negative poles and record the potential difference between the two poles. Unipolar leads are aV_R_, aV_L_, and aV_F_, which, unlike bipolar leads, measure the potential difference between a positive point and the average potential of the other two points [37].

**Fig. 2:**
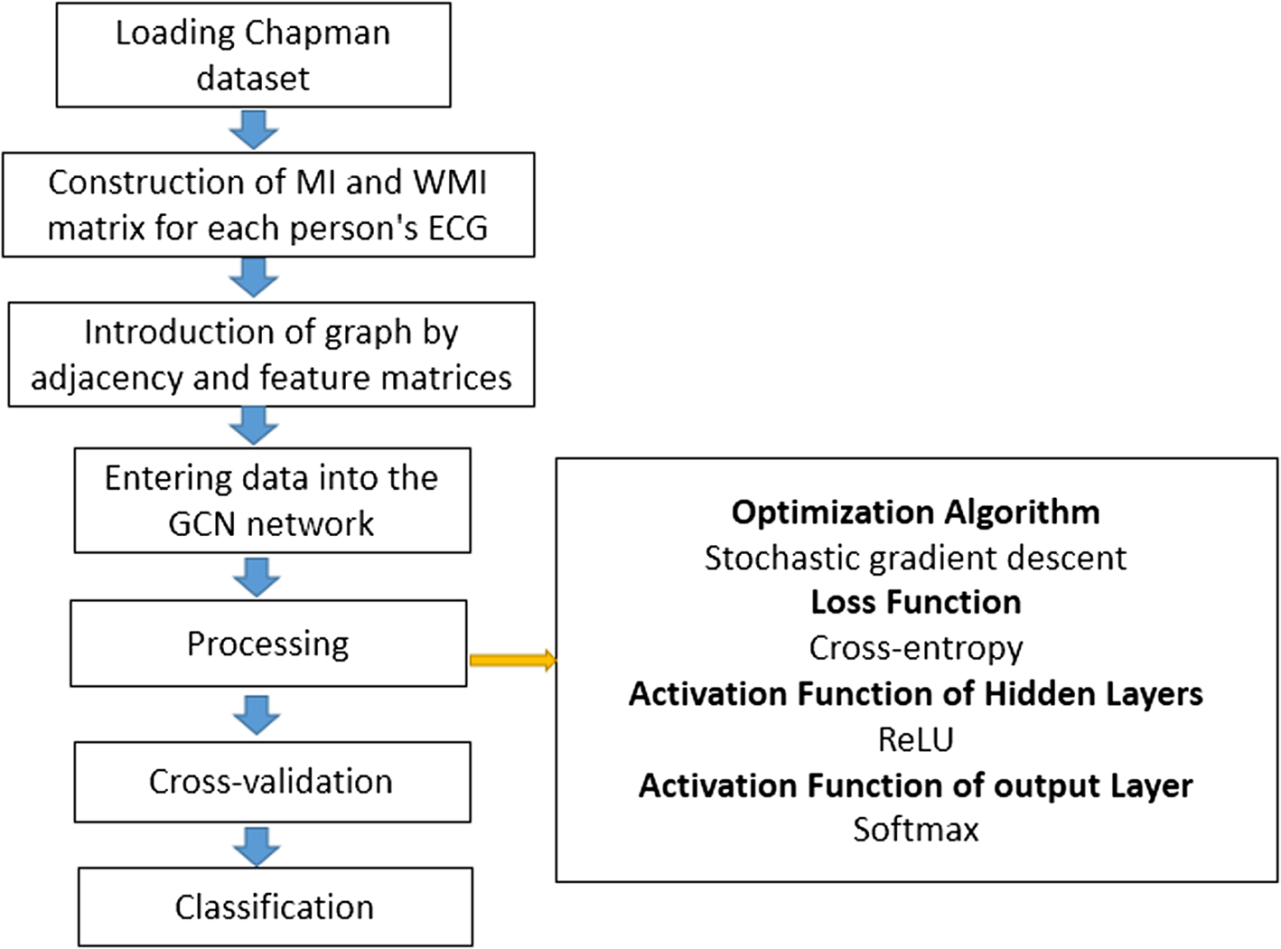
General block diagram for the classification process

In this study, the proposed new GCN-WMI network structure was implemented, adjusted, and applied to detect and classify the type of arrhythmia. Figure 2 shows a general block diagram for the classification process.

Considering that one of the factors to increase the accuracy of learning in neural networks is the volume of data, the latest ECG data set with more than 10,000 cases was used in this research.

In previous studies, the relationship between cardiac leads has not been investigated by a suitable and relevant statistical index. In this study, for the first time, we proposed a suitable statistical index to evaluate the relationship between cardiac leads used it in the GCN network to detect arrhythmia.

It is very important, from a clinical point of view, to diagnose all types of arrhythmias with accuracy and speed by ECG tools, which are cheap and available. The practical goal of this research is to increase the accuracy of this diagnosis and the practical use of this network in hospital systems.

### Innovative aspects of this study

- Each person’s ECG information was defined in the form of a chart.
- A new structure (WMI) was introduced for ECG data.
- For the first time, the proposed new GCN-WMI network structure was used to detect and classify people’s arrhythmia types.

## Methods

### ECG data

**Fig. 3:**
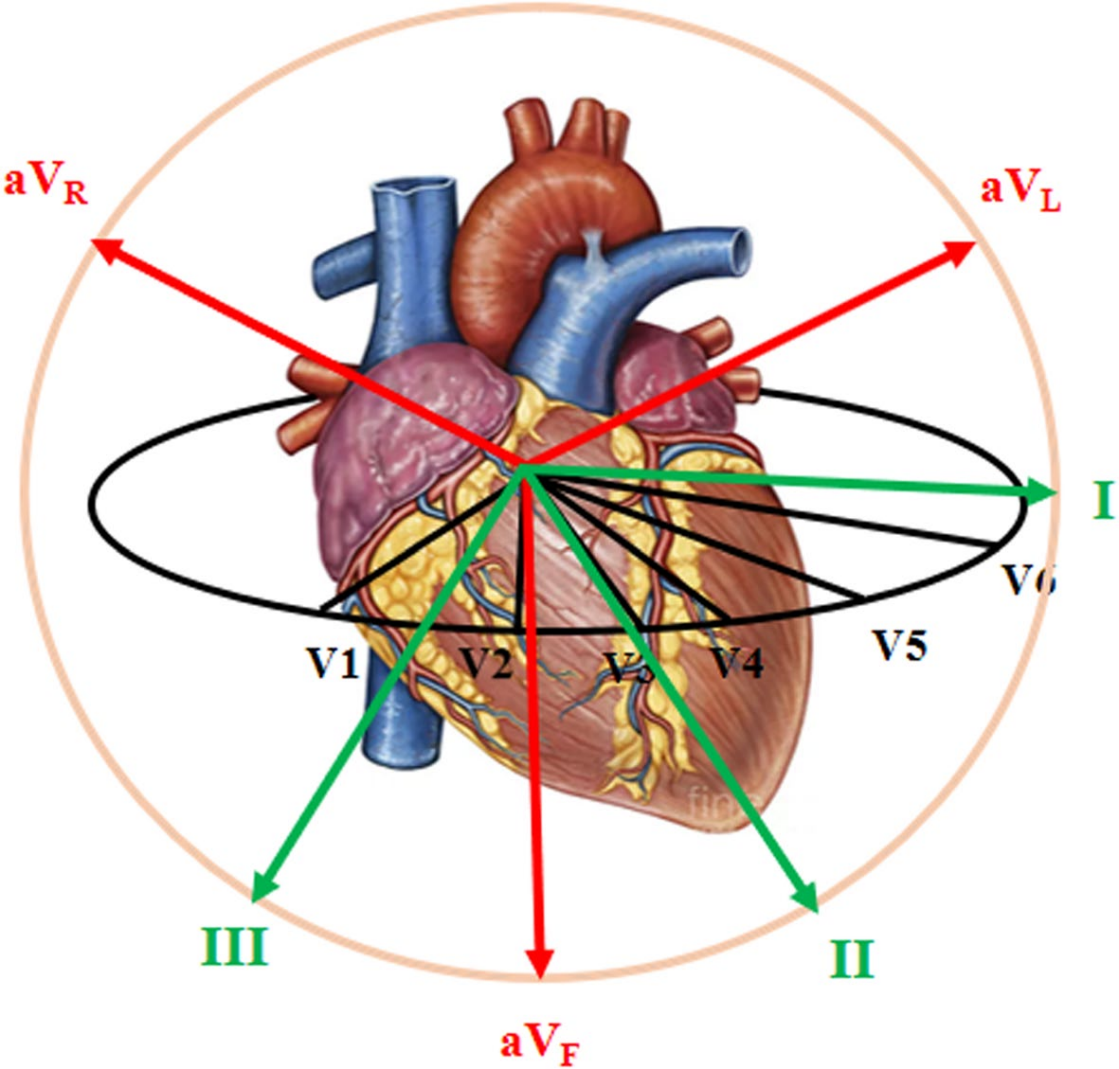
12 different angles of measuring electric potential in ECG

The 12-Lead ECG is one of the most widely used diagnostic tools for heart diseases and types of arrhythmias. In a 12-lead ECG machine, over a period of time (usually ten seconds), the total value of the electrical potential of the heart is recorded from twelve different angles by attaching ten electrodes to the chest and limbs [38]. Figure 3 shows different angles of electrical measurement of the heart in ECG.

**Fig. 4:**
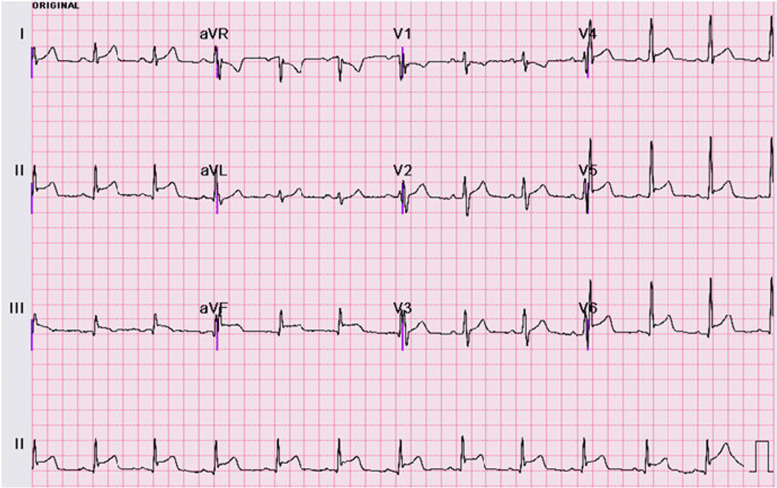
A 12-lead electrocardiogram on admission revealing a normal sinus rhythm with non-specific ST elevation (I, II, III, aVF, and V2-V6 leads)

The electrical waves of the heart are drawn by the ECG machine at a regular rate on a special paper. Diagnosing the type of cardiac arrhythmia according to the drawn shapes requires expertise. Figure 4 shows a 12-lead ECG with a normal rhythm.

**Fig. 5:**
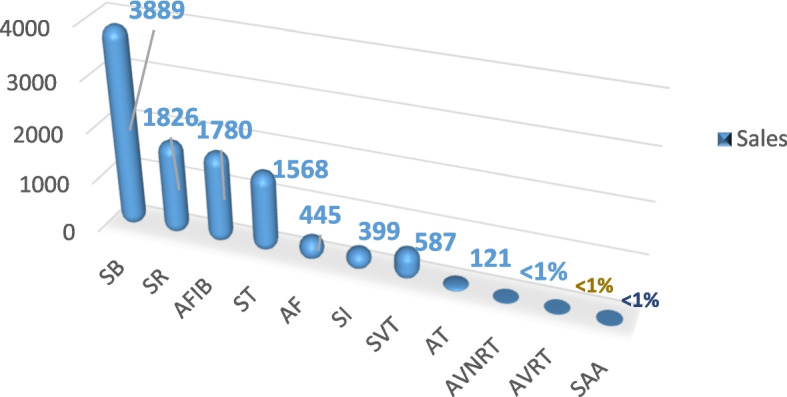
The frequency of rhythms

In this study, the 12-Lead ECG data of 10,646 people were used. The data were related to the research database under the supervision of Chapman University and People’s Hospital (Zhaiyang Medical University School of Medicine, Shaoxing Hospital) [5]. The sampling rate of these ECGs was 500 Hz for 10 s. This means that 5000 samples per lead are available per person. The arrhythmia type of these ECGs was labeled by professional experts. Of the subjects, 17% had normal sinus rhythm and 83% had at least one abnormality. Figure 5 shows the frequency diagram of rhythms.

In the present study, data related to seven types of arrhythmias were used, and data related to four types of arrhythmias, including AT, AVNRT, AVRT, and SAA, were excluded because the frequency ratio of these four types of arrhythmias was less than 2% of the total frequencies. When there is a class imbalance in the training data, learning is done more for high-volume classes, and as a result, the classification error is greater for items belonging to the minority group than for items belonging to the majority group [39].

### Structure between data

Clinically, there is a connection between the ECG leads, because the ECG acts like a camera that observes the heart’s function from different angles (leads). In this study, the MI index was used to introduce the new structure. The available data for each lead is a time series, so the use of Pearson and Spearman correlation coefficients is not allowed due to the non-establishment of the iid assumption, and the MI index measures the linear and non-linear relationship between two-time series. The MI index can be measured for the connection between two leads, so a 12 × 12 matrix shows the connection between 12 ECG leads.

### Mutual information

Mutual Information (MI) originates from the definition of entropy. The joint entropy of a pair of random variables (X, Y) expresses uncertainty about the combination of these variables.

$$\text{H}\left[\text{X, Y}\right] = -\sum\limits_{x\in X,y\in Y}\text{Pr}\left[\mathrm{X} = \text{x, Y} = \text{y}\right]\cdot \log \text{Pr} \left[\text{X} = \text{x, Y} = \text{y}\right]$$

The conditional entropy of a random variable X with respect to another variable Y expresses the uncertainty of X that remains after Y is known:

$$\mathrm H\left[\mathrm X\vert\mathrm Y\right]=-\sum\limits_{x\in X,y\in Y}\text{Pr}\left[\mathrm X=\mathrm x,\;\mathrm Y=\mathrm y\right]\cdot\mathrm{logPr}\left[\mathrm X=\mathrm x\vert\mathrm Y=\mathrm y\right]$$

MI is a measure of dependence between two variables. This is the amount of information obtained by observing Y from X. In discrete mode, the mutual information of two variables X and Y is obtained as follows:

$$\mathrm I\left(\mathrm X;\;\mathrm Y\right)=\sum\limits_{x\in X,y\in Y}\text{Pr}\left[\mathrm X=\mathrm x,\;\mathrm Y=\mathrm y\right]\cdot\log\left(\frac{\text{Pr}\left[\mathrm X=\mathrm x,\;\mathrm Y=\mathrm y\right]}{\text{Pr}\left[\mathrm X=\mathrm x\right]\cdot\text{Pr}\left[\mathrm Y=\mathrm y\right]}\right)$$

MI can also be written in terms of conditional probability:

$$\mathrm I\left(\mathrm X;\;\mathrm Y\right)=\sum\limits_{x\in X}\Pr\left[\mathrm X=\mathrm x\right]\sum\limits_{y\in Y}\Pr\left[\mathrm Y=\mathrm y\vert\mathrm X=\mathrm x\right]\cdot\log\left(\frac{\Pr\left[\mathrm Y=\mathrm y\vert\mathrm X=\mathrm x\right]}{\Pr\left[\mathrm Y=\mathrm y\right]}\right)$$

This relationship can be written in terms of Shannon’s entropy as follows:

$$\begin{array}{c}\mathrm I\left(\mathrm X;\;\mathrm Y\right)=\mathrm H\left[\mathrm X\right]-\mathrm H\left[\mathrm X\vert\mathrm Y\right]\\=\mathrm H\left[\mathrm X\right]+\mathrm H\left[\mathrm Y\right]-\mathrm H\left[\mathrm X,\;\mathrm Y\right]\\=\mathrm H\left[\mathrm X,\;\mathrm Y\right]-\mathrm H\left[\mathrm X\vert\mathrm Y\right]-\mathrm H\left[\mathrm Y\vert\mathrm X\right]\end{array}$$

**Fig. 6:**
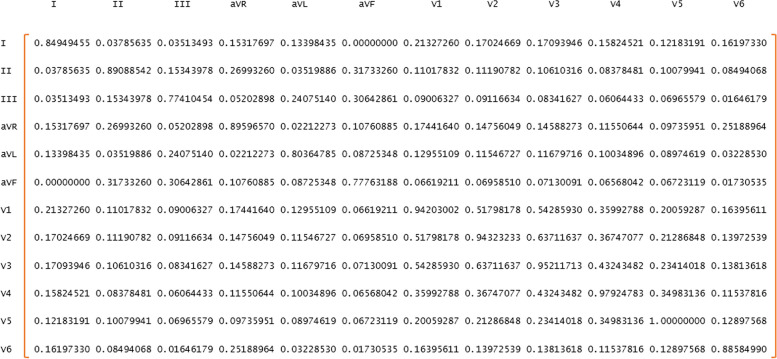
A 12 × 12 matrix of mutual information between cardiac leads for one person

MI is always greater than or equal to zero and will be zero if X and Y are independent. For the continuous state, all relations can be expressed by integrals [40–42]. In this study, the MI_N×N_ matrix was calculated for 12 ECG leads by R software. That is, based on each person’s ECG, MI is measured between 12 heart leads (each lead contains 5000 measured values). Figure 6 shows an example of this matrix.

### The new structure of WMI

**Fig. 7:**
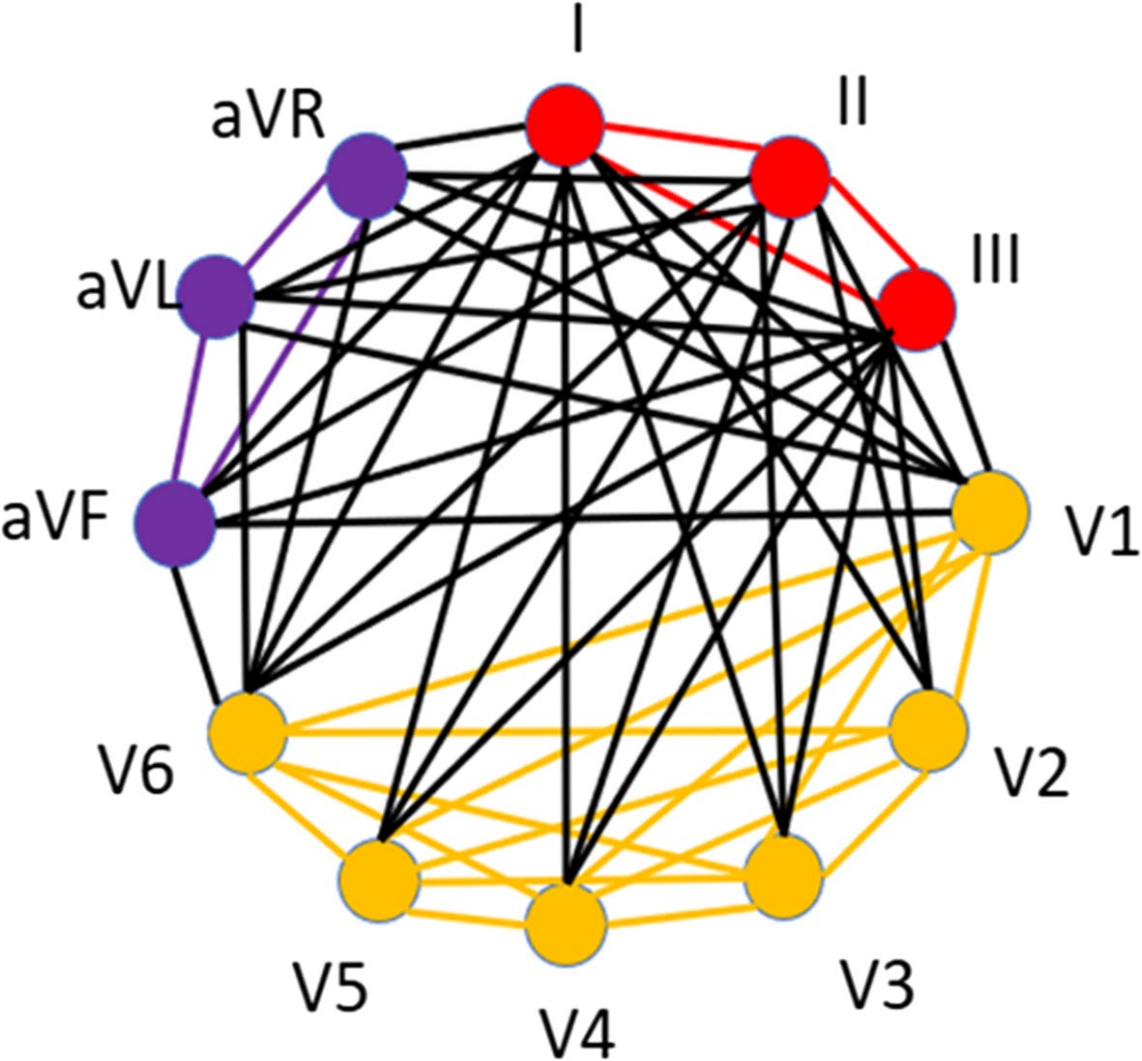
The 12-Lead ECG with weighted mutual information structure

According to the poles of the electrocardiogram, in this study, the MI matrix was weighted. Precordial leads, including V1, V2, V3, V4, V5, and V6, unipolar limb leads, including aVR, aVL, and aVF, and bipolar limb leads, including I, II, and III, are considered three clusters (Fig. 7).

In this way, by the following indicator function, more weight is assigned to the mutual information between the leads located in a cluster, which indicates the importance of the polarity of the electrocardiogram leads in the data structure.

$$A\left(i, j\right)={MI}_{12\times 12} \times \left[1+r\left({Q}_{i}, {Q}_{j}\right)\right]$$

$$r\left(Q_i,Q_j\right)=\left\{\begin{array}{ccc}1&if&Q_i=Q_j\\0&if&Q_i\neq Q_j\end{array}\right.$$

Q is the lead pole symbol. i = 1, 2, …, 12 and j = 1, 2, …, 12 represent the rows and columns of the MI matrix. That is, if each of the two ECG leads is placed in the same pole (cluster), the MI index will be calculated between them doubles, otherwise, it does not change.

**Fig. 8:**
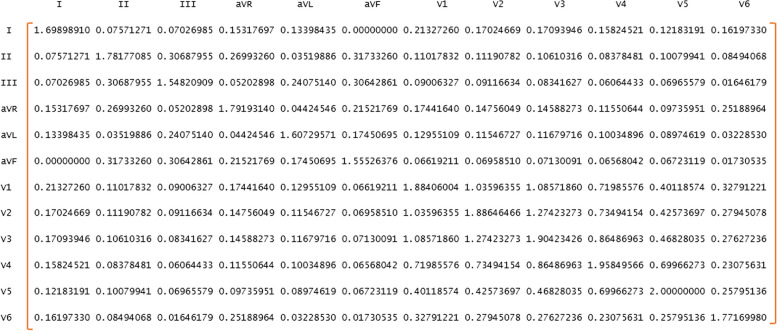
A 12 × 12 matrix of weighted mutual information

To clarify the discussion, by this proposed structure, the connection (MI) between the leads, marked with the same color in Fig. 6 (having the same polarity), is doubled, but the connection between the leads whose colors are different does not change, because clinically, leads that are in the same polarity are more similar. Figure 8 shows an example of a WMI matrix.

### GCN

By displaying data in graph form, structural information can be encoded to model the relationships between data and provide better insight into the underlying data [34]. An undirected, connected, weighted graph is denoted by G=(V, E, W) with a vertex set V with |V|=N, an edge set E, and a weighted adjacency matrix W. If there is an edge between two vertices i, j, e=(i, j), its weight is W_i, j_, otherwise W_i, j_=0 [43].

Graph Neural Network (GNN) forms the basis of all types of graph networks. This network was introduced to the literature in this field in 2008 based on graph theory. Graph Convolutional Network (GCN) is one of the most famous graph networks that mainly uses the combination of Fourier transform and Taylor expansion formula to improve the filter performance. This network learns features by examining adjacent nodes and performs a mathematical operation called convolution on the graphs [44]. The main purpose of this type of network can be the classification of graphs, nodes, or connections [45]. GCNs themselves can be divided into two main categories of algorithms, Spectral Graph Convolutional Networks (SGCNs) and Spatial Graph Convolutional Networks (SGCNs). In this study, Spatial Graph Convolutional Networks were used to classify graphs.

#### Graph convolution

The data of a graph is related to the information on the edges and vertices (nodes) of that graph. The convolution filtering method is used to process and learn this information. In this method, both edge information and vertex information are considered for the filter. A convolutional filter method is a spatial approach that follows a local neighborhood graph filtering strategy.

The graph convolution operation is performed using a polynomial which is formed based on the adjacency matrix of the graph. Using this polynomial, the features of each vertex (node) are combined with the features of the neighboring vertices.

1 $$H = h_{0}I+h_{1}A^{1}+h_{2}A^{2}+h_{3}A^{3}+\cdots+h_{k}A^k$$

These polynomials can be considered equivalent to filters in CNN and hi coefficients as weights. In Eq. 1, the largest power of the polynomial, K, determines the number of neighborhood steps from a vertex, so the filter matrix is obtained as *H* ∈ ℝ_*N*_ × _*N*_. The convolution of vertices V with filter H is a matrix multiplication shown below, where *V*_*out*_, *V*_*in*_ ∈ ℝ_*N*_. *V*_*in*_ is the initial vertex matrix and *V*_*out*_ is the vertex matrix after the filter operation.

$${V}_{out}=H{V}_in$$

### The proposed architecture for the GCN network algorithm with three steps

In the first step, in order to prepare the graph, the active and inactive nodes are equalized and considered aligned. However, a separate entry will be regarded as a label next to it. The output of this step will be included in the next step as graph Z_0_.

**Fig. 9:**
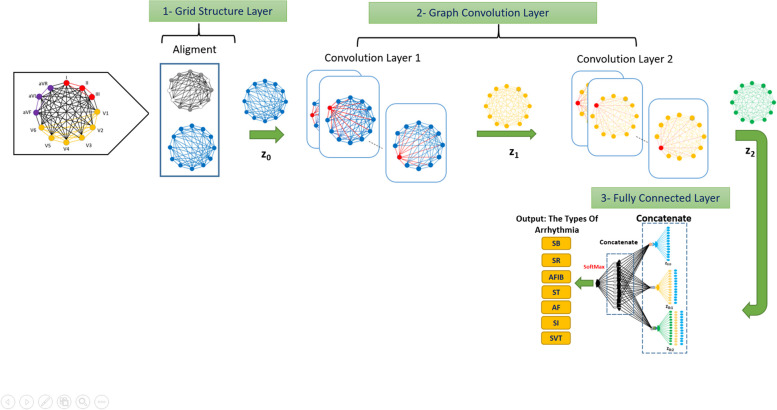
The network architecture for GCN with two layers of convolution

Then, in the second step, in several layers, the convolution operation will be performed on the graph. In this way, the desired filter will be applied to each node by considering the neighboring edges. Figure 9 shows this architecture for a GCN network with two convolution layers and graphs with 12 nodes. In the convolution layers, the nodes that have undergone operations are shown in red at each step. Z_1_ is the output of the first layer of convolution, which is considered the input of the second layer, and Z_2_ is created as the output of the second layer.

In the third step, using all the graphs created in the previous steps and their permutations from Z_0_ to Z_2_, several linear vectors are created, which are called linear features. This operation is performed in two stages of the Concatenate operation. The output of these two stages is fed into the fully connected neural network at the last level. Finally, by using the network known as Softmax, the decision about the response variable is made.

### Methodology

This study aimed to set up a GCN network with a new structure to automatically detect the type of arrhythmia in people based on 12-Lead ECG. Therefore, the data available in the Chapman research database were used, which includes the 12-Lead ECG information of 10,494 people. In each lead, the electrical potential of the heart is measured 5000 times (every ten seconds). That is, the feature matrix for each person is a 5000 × 12 matrix.

Zheng et al. collected and presented these data and performed the pre-processing operation. For this purpose, noise reduction operations were performed. Sources of noise pollution include power line interference, motion artifacts, electrode contact noise, baseline drift, muscle contraction, and random noise. Zhang et al. [5] proposed and implemented a sequential denoising approach to remove noise from raw ECG data. In convolution-based networks, the feature selection operation is performed at the same time as the network training, and there is no need to pre-process the complexity of the data.

In the next step, the 12 × 12 MI matrix was calculated by R software to determine the relationship between the pairs of leads. Then, the new structure introduced in this article was applied to this matrix, and the WMI 12 × 12 matrix was formed. GCN was set with adjacency matrices, WMI_12 × 12_, MI_12 × 12_, and identity matrix (in this case the relationship between leads is ignored). At first, suitable GCN networks were selected in terms of the number of layers and parameters, with all three types of adjacency matrices. Next, all individuals were classified by the selected networks. Finally, to choose the best adjacency matrix, the performance of the three networks was compared with the mentioned adjacency matrices.

The cross-validation method with 4 folds was used to evaluate the model. To select the number of network layers, GCN was trained with 5, 10, and 15 layers. To select the most suitable accuracy network, the three mentioned networks were compared two by two by independent t-test.

Network performance evaluation criteria included accuracy, specificity, precision, and sensitivity in test sets. Also, to evaluate the classification of all people by the selected networks, a confusion matrix was prepared.

Considering True Positive (TP), True Negative (TN), False Positive (FP) and False Negative (FN), the calculation formula of these criteria are as follows:

$$\begin{array}{l}\mathrm{Accuracy}=\left(\mathrm{TP}\:+\:\mathrm{TN}\right)/\mathrm{Total}\\\mathrm{Sensitivity}=\mathrm{TP}/\left(\mathrm{TP}+\;\mathrm{FN}\right)\\\mathrm{Specificity}=\mathrm{TN}/(\mathrm{FP}+\mathrm{TN})\\\mathrm{Precision}=\mathrm{TP}/(\mathrm{TP}+\mathrm{FP})\end{array}$$

## Experimental results

**Fig. 10:**
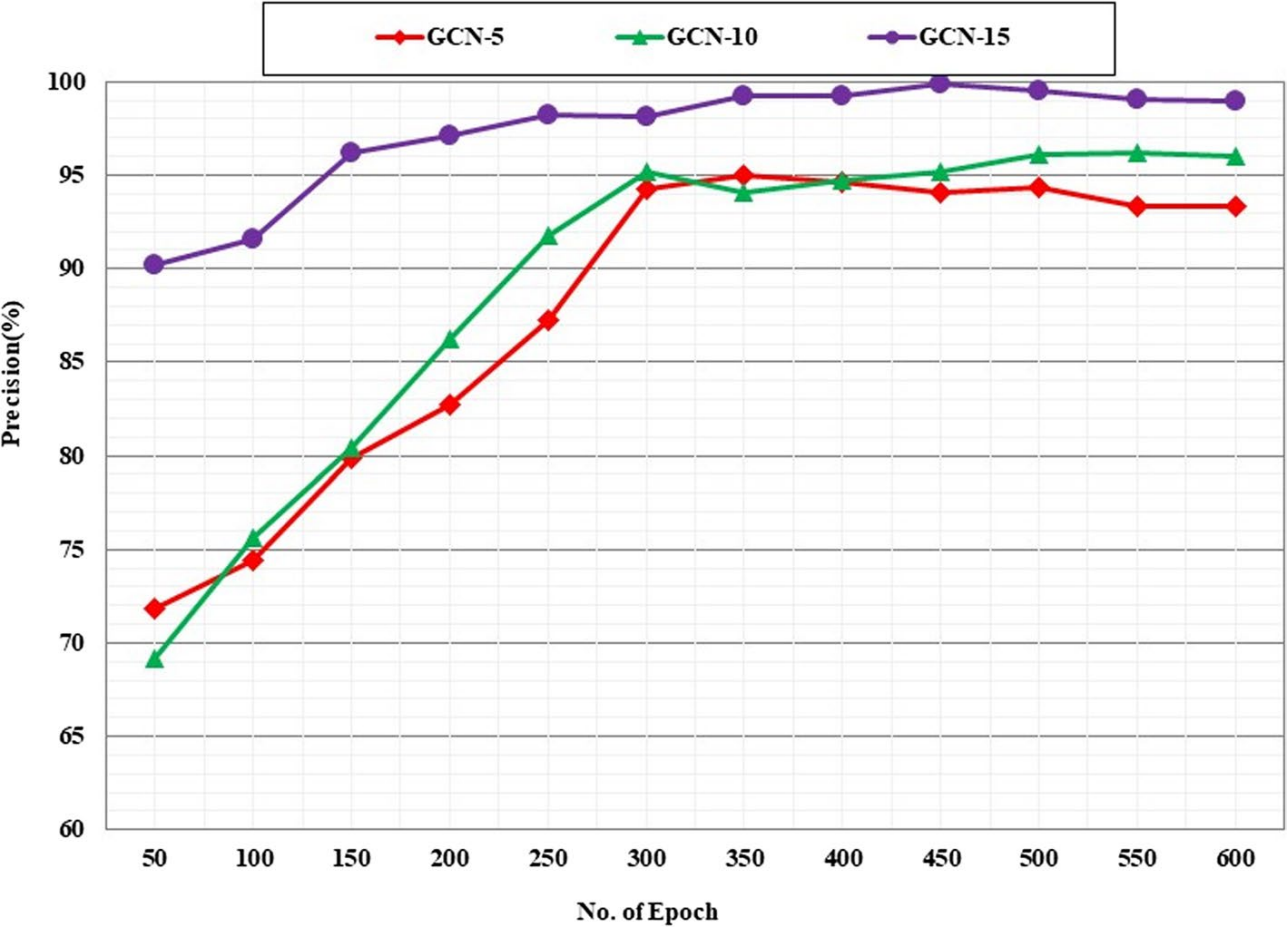
Learning curve to compare the cross-validation precision of GCN-WMI with 5, 10 and 15 layers

WMI matrices were calculated using R software. The GCN network was set up and implemented with the WMI adjacency matrix with 5, 10, and 15 layers. The adjusted parameters are shown in Table 1. The learning curve to compare the cross-validation precision of GCN-WMI with 5, 10, and 15 layers showed that the accuracy of GCN-WMI with 15 layers was higher (Fig. 10).

**Table 1:** The parameter of the GCN-WMI network

Parameter	Value
**Learning Rate**	0.02
**Epochs**	600
**Hidden2**	5
**Hidden2**	10
**Hidden2**	15
**Dropout**	0.2
**Weight Decay**	0.0005
**Early Stopping**	10

**Fig. 11:**
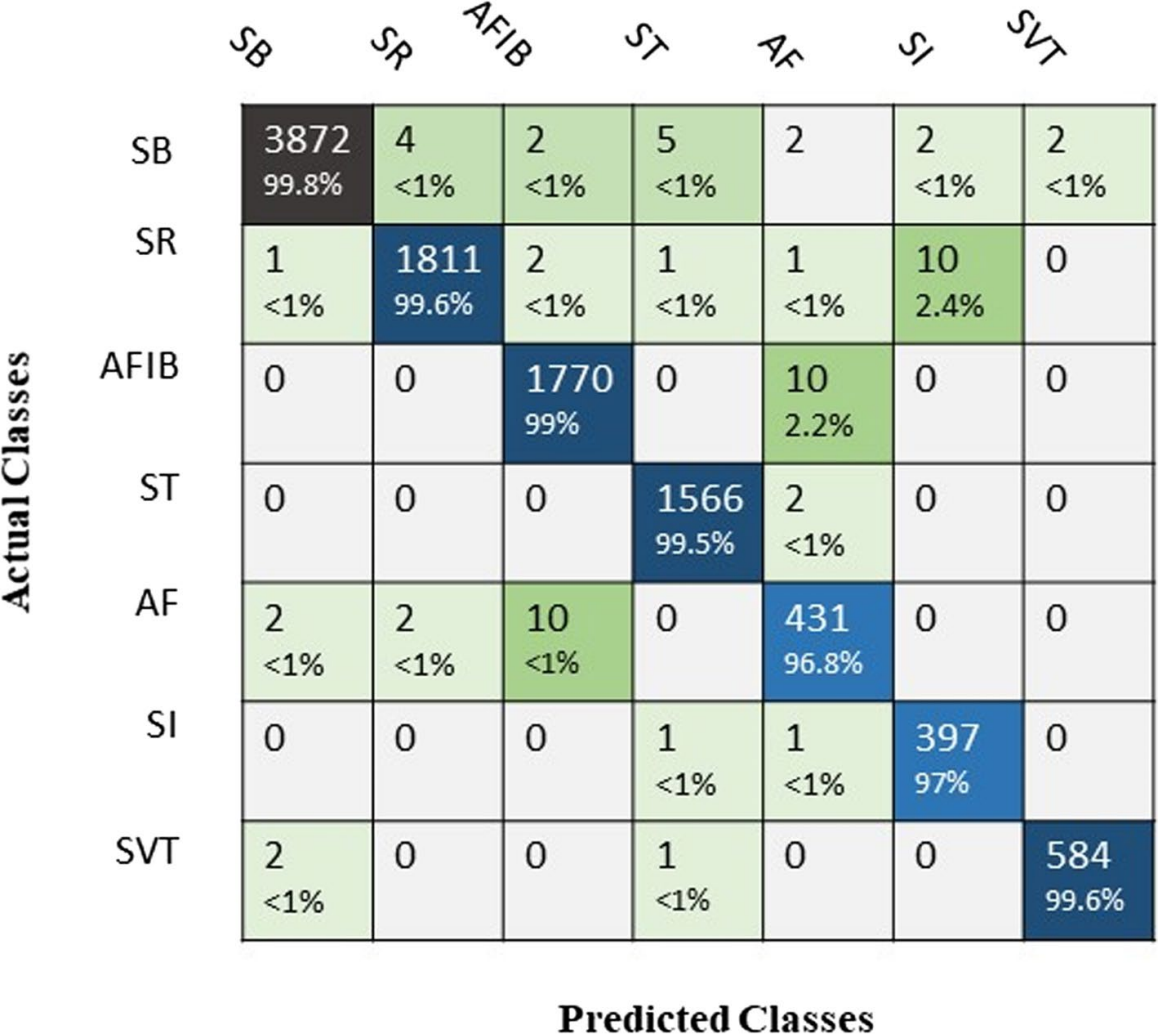
Confusion matrix for all records using GCN-WMI

It is obvious that adding more layers increases the accuracy of feature extraction, but we should consider the possible overfitting by adding layers and be aware of possible overfitting. Considering the accuracy and other predictive performance values of GCN-WMI with different layers and the slight increase in computational cost, GCN-WMI with 15 intermediate layers was selected and configured using cross-validation. Therefore, the GCN-WMI network was set with 15 layers, and the arithmetic type of all data (10,494) was predicted by this network. Confusion matrix results showed that 99.8% of GCN-WMI network predictions were correct for SB arrhythmia. The percentages were 99.6%, 99%, 99.5%, 96.8%, 97%, and 99.6% for SR, AFIB, ST, AF, SI, and SVT arrhythmias, respectively (Fig. 11).

The results of network evaluation indices showed that the said network had the highest sensitivity (99.87%) for SB arrhythmia detection, the highest precision (99.87%) for ST detection, the highest specificity (99.95%) for SI & SVT detection, and the highest accuracy (99.95%) for SVT (Table 2).

**Table 2:** The performance values of all records for each class separately using GCN-WMI

	Sensitivity(%)	Precision(%)	Specificity(%)	Accuracy(%)
SB	99.87	99.51	99.71	99.77
SR	99.67	99.18	99.69	99.80
AFIB	99.05	99.44	99.77	99.74
ST	99.50	99.87	99.89	99.90
AF	96.42	96.63	99.75	99.70
SI	97.06	99.50	99.95	99.86
SVT	99.66	99.15	99.95	99.95

Finally, three GCN-WMI, GCN-MI, and GCN-Id networks with 15 layers were compared with WMI, MI, and identity adjacency matrices, respectively. The sensitivity, precision, specificity, and accuracy of the GCN-WMI network with 98.74%, 99.08%, 99.97%, and 99.82% were more than those of the other two networks (Table 3). It seems that the structure introduced in this article has been effective in increasing the accuracy of detecting and classifying the type of arrhythmia.

**Table 3:** The performance values for all records using GCN-WMI, GCN-MI and GCN-Id

		Sensitivity	Precision	Specificity	Accuracy
Overall	*GCN-WMI*	**0.9874 ± 0.63**	**0.9908 ± 0.64**	**0.9997 ± 0.35**	**0.9982 ± 0.38**
	*GCN-MI*	**0.9845 ± 0.55**	**0.9789 ± 0.52**	**0.9985 ± 0.37**	**0.9971 ± 0.41**
	*GCN(Identity)*	**0.6824 ± 0.51**	**0.7283 ± 0.44**	**0.9524 ± 0.48**	**0.9268 ± 0.48**

## Discussion

This study aimed to detect the type of arrhythmia in people through a new GCN-WMI network, according to the type of ECG data and the relationship between heart leads. For this purpose, using the Chapman dataset, 7 types of arrhythmias were recognized and classified by GCN-WMI with 15 layers. Several studies have used this data set to diagnose cardiac arrhythmia and have diagnosed and classified 7 types of arrhythmia by the proposed methods. Yıldırım et al. introduced the DNN method to detect the type of cardiac arrhythmia with an accuracy of 92.24% [46]. Meqdad et al. used CNN Trees and Meta CNN Trees methods and detected the type of cardiac arrhythmia, with accuracy rates of 97.60% and 98.29%, respectively [47, 48]. Mehari et al. also introduced the Single Classifier method for this purpose, whose accuracy value was equal to 92.89% [49]. Rahul et al. proposed a 1-D CNN method with an accuracy of 94.01% [50], Kang et al. employed the RNN method with an accuracy of 96.21% [51], Domazetoski et al. applied XGBoost method with an accuracy equal to 89.40% [52], and Sepahvand et al. introduced two methods, Teacher model and Student model, with accuracies of 98.96% and 98.13%, respectively [53]. The accuracy of the GCN-WMI method (99.82%) that was introduced in the present study was higher than those of the aforementioned methods (Table 4).

**Table 4:** Performance comparison of the proposed method with other state-of-the-art using the Chapman dataset

Ref	Study	Dataset	Num. of subjects	Year	Method	Classes	Performance
[46]	Yildirim et al.	Chapman	10,646	2020	DNN	7	Acc = 92.24%
[47]	Meqdad et al.	Chapman	10,646	2022	CNN Trees	7	Acc = 97.60%
[48]	Meqdad et al.	Chapman	10,646	2022	Meta CNN Trees	7	Acc = 98.29%
[49]	Mehari et al.	Chapman	10,646	2022	Single Classifier	7	Acc = 92.89%
[50]	Rahul et al.	Chapman	10,646	2022	1-D CNN	7	Acc = 94.01%
[51]	Kang et al.	Chapman	10,646	2022	RNN	7	Acc = 96.21%
[52]	Domazetoski et al.	Chapman	10,646	2022	XGBoost	**-**	Acc = 89.40%
[53]	Sepahvand et al.	Chapman	10,646	2022	Teacher model	**7**	Acc = 98.96%
					Student model	**7**	Acc = 98.13%
	**Proposed**	**Chapman**	**10,646**	**2022**	**GCN-WMI**	**7**	**Acc = 99.82%**

In several other studies, new methods have been proposed to detect cardiac arrhythmia, and the accuracy of none of these methods has been higher than that of our proposed method. In a similar work by Jiang et al. [54], two networks CNN and then GCN were used to detect multiple heart disorders. They used GCN to determine the relationship between arrhythmia classes when more than one cardiac disorder was present during ECG signal collection and used binary cross-entropy loss for correlation between labels. However, the structure between the heart leads was ignored. This can be another strength of our proposed method, where each ECG is defined as a graph and directly expressed by GCN to detect and classify the type of arrhythmia.

Shaker et al. proposed a GAN method, and the accuracy of this method for classifying MIT-BIH data into 15 classes was equal to 98.30% [31]. In their study, Zhao and Ban proposed the CNN + ELM method to classify MIT-BIH data into 4 classes. This method is a combination of Convolutional Neural Network (CNN) and Extreme Learning Machine (ELM), with an accuracy of 97.5% [30]. Other researchers have also proposed methods for arrhythmia classification on the MIT-BIH dataset, which includes 48 ECG data. Gao et al. proposed the LSTM, FL method to detect 5 types of arrhythmia, the accuracy of which was equal to 99.26% [55]. Oh et al. also proposed the Modified U-net method for the automatic detection and encoding of 5 types of ECG arrhythmias, and the accuracy of this method was equal to 97.32% [56]. Li et al. [57] used the ResNet method with 99.38% accuracy, Yildirim et al. [58] used the CNN method with 91.33% accuracy, Xu et al. [59] used the DNN method with 93.1% accuracy, and Acharya et al. [60] suggested CNN method with 94.03% accuracy.

## Conclusion

The aim of this study was the simultaneous use of all ECG leads and the connection between them. It is highly important to detect the right relationship between the leads and consequently form the right structure. It seems that the proposed structure for communication between ECG leads was effective in this study. The results showed that the proposed method in this research is more accurate than the ones reported in the above studies. The present study is the first study that focuses on the relationship between cardiac leads. It has improved the diagnosis of arrhythmia in people by presenting a new structure and using it in the GCN network. The proposed models in different studies can provide a basis for developing future diagnostic applications. Therefore, it is important to increase the accuracy of models with new suggestions.

### Limitation

The implementation of DL methods, including the method proposed in this article, requires strong systems.

Another limitation of using the proposed method is the limitation of its application to wearable ECG data because this type of ECG is a single signal.

## Data Availability

Data are available at https://figshare.com/collections/ChapmanECG/4560497/2 (accessed on 25 September 2020).
